# Jujube Polysaccharide Promotes Neuroprotection and Longevity in *Caenorhabditis elegans* Through Oxidative Stress Resistance and Stress-Response Signaling

**DOI:** 10.3390/ijms27114727

**Published:** 2026-05-24

**Authors:** Zhiying Hou, Ayaz Ahmed, Jiayin Wang, Meng Sun, Fengzhong Wang, Qiong Wang

**Affiliations:** 1 Institute of Food Science and Technology, Chinese Academy of Agricultural Sciences, Beijing 100193, China; 2 National Nanfan Research Institute (Sanya), Chinese Academy of Agricultural Sciences, Sanya 572024, China

**Keywords:** jujube polysaccharide, Parkinson’s disease, *Caenorhabditis elegans*, oxidative stress, neuroprotection

## Abstract

Parkinson’s disease (PD) involves oxidative stress, proteotoxic aggregation, and neurotransmitter dysfunction, yet current therapies remain largely symptomatic. This study investigated whether Jujube polysaccharides (ZJP), a food-derived polysaccharide, confer neuroprotective and anti-aging benefits in *Caenorhabditis elegans*. ZJP was characterized for physicochemical features, antioxidant capacity, and in vivo safety. Effects were evaluated in wild-type N2 and PD models by measuring lifespan, locomotion, pharyngeal pumping, chemotaxis, α-syn::YFP fluorescence intensity, dopaminergic neuron integrity, adenosine triphosphate (ATP), reactive oxygen species (ROS), superoxide dismutase (SOD), catalase (CAT), malondialdehyde (MDA), and lipofuscin. Stress resilience was assessed under heat (37 °C) and H_2_O_2_ exposure. RT-qPCR profiled genes related to stress responses and neurotransmission. ZJP showed no detectable toxicity at tested doses. ZJP extended mean lifespan in N2 (10.3–14.1%) and NL5901 (9.1%), improved locomotion, pharyngeal pumping, and chemotaxis, reduced lipofuscin (26.8–50.6%), and increased survival under heat (23.6%) and oxidative stress (38.1%). In PD models, ZJP reduced α-syn::YFP fluorescence by up to 54.9%, protected dopaminergic neurons, and increased ATP. It also lowered ROS and MDA levels while raising SOD and CAT activities. Gene expression changes were associated with enhanced oxidative stress resistance and with altered expression of genes involved in SKN-1/DAF-16-related stress-response signaling. These findings provide preliminary evidence that ZJP may promote longevity, stress resilience, and neuroprotection in *C. elegans* models of PD, supporting its potential as a candidate for further investigation in neuroprotection.

## 1. Introduction

The accelerating global aging population has made aging-related functional decline and neurodegenerative diseases (NDs) a major public health concern. Parkinson’s disease (PD) ranks second among neurodegenerative disorders in frequency, and its prevalence rises with age [1,2]. The cardinal motor symptoms, i.e., resting tremors, bradykinesia, rigidity, and postural instability, collectively contribute to substantial functional impairment with diminished quality of life and a shortened lifespan [3,4]. The neuropathological hallmarks of PD consist of dopaminergic neuron loss in the substantia nigra, alongside accumulation of misfolded α-synuclein into Lewy bodies [5]. Oxidative stress is a widely recognized shared pathological mechanism underlying both aging and PD [6]. During aging, the accumulation of reactive oxygen species disrupts redox homeostasis and accelerates functional decline. In PD, dopaminergic neurons are vulnerable to oxidative injury due to their high metabolic demand and limited endogenous antioxidant capacity, resulting in mitochondrial dysfunction, inflammation, and progressive neurodegeneration [7,8,9]. Although levodopa and dopamine receptor agonists remain first-line therapeutics, they offer only symptomatic relief without halting disease progression, and long-term use is associated with motor fluctuations and drug resistance [10]. Thus, safe, sustainable, multi-target interventions capable of regulating oxidative stress, inflammation, and proteostasis are urgently needed.

Food-derived bioactive polysaccharides have gained considerable attention due to their favorable biocompatibility, low toxicity, and suitability for long-term consumption [11]. Polysaccharides from traditional dietary sources such as *Lycium barbarum*, *Ganoderma lucidum*, and *Codonopsis pilosula* have been reported to exhibit significant antioxidant, anti-inflammatory, and neuroprotective effects [12,13,14]. For example, *Lycium barbarum* polysaccharides improved dopaminergic function and reduced oxidative damage in MPTP-induced PD models, while other natural polysaccharides mitigate neurodegeneration by modulating oxidative stress and inflammation [15,16]. However, whether dietary polysaccharides can simultaneously modulate α-syn aggregation, neurotransmitter homeostasis, and overall health outcomes in the context of PD pathology remains largely unexplored.

*Ziziphus jujuba* Mill. (jujube) is a widely consumed edible-medicinal fruit rich in polysaccharides, flavonoids, and saponins [17]. Jujube polysaccharides (ZJP) exhibit antioxidant, immunomodulatory, and anti-inflammatory functions [18,19,20]. Our recent findings indicate that ZJP ameliorates cognitive impairment and restores intestinal barrier integrity in chronic stress models. However, current studies on ZJP have primarily focused on general antioxidant and anti-inflammatory effects, with limited investigation into its role in neurodegenerative disease models. In particular, whether ZJP can simultaneously modulate α-synuclein aggregation, dopaminergic neuron integrity, and stress-response signaling in vivo remains unclear.

*Caenorhabditis elegans* (*C. elegans*) has emerged as a robust and genetically tractable model for studying aging and neurodegeneration due to its short lifespan, transparent nervous system, and highly conserved pathways implicated in human PD [21,22,23]. Transgenic models such as NL5901 (α-syn::YFP) and BZ555 (GFP-labeled DA neurons) enable direct quantification of α-syn aggregation, dopamine (DA) neurotoxicity, mitochondrial dysfunction, and locomotor decline. We therefore hypothesized that ZJP exerts multitarget neuroprotection by enhancing endogenous antioxidant defenses, mitigating α-syn proteotoxicity, and improving sensory–motor function. This study systematically evaluated the behavioral, biochemical, and molecular effects of ZJP in aging and PD-related *C. elegans* models, providing experimental evidence to support ZJP as a functional food ingredient for neuroprotective health management.

## 2. Results

### 2.1. Safety Evaluation of ZJP

**Figure 1:**
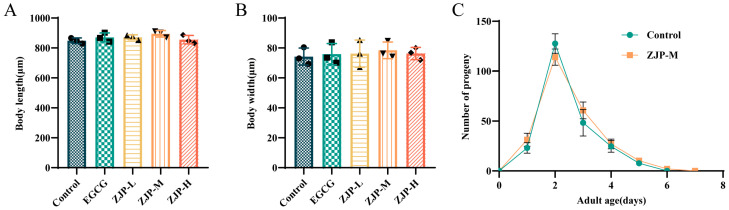
Safety evaluation of Jujube polysaccharides (ZJP) in *Caenorhabditis elegans* (*C. elegans*). (**A**) Body length (*n* = 30, mean ± SD). (**B**) Body width (*n* = 30, mean ± SD). (**C**) Brood size (*n* = 10, mean ± SD). Control, untreated; EGCG, epigallocatechin gallate, a green tea polyphenol used as the positive control at 500 μM; ZJP-L, ZJP-treated group at 200 μg/mL; ZJP-M, ZJP-treated group at 400 μg/mL; ZJP-H, ZJP-treated group at 800 μg/mL. Experiments were performed in three independent replicates.

Toxicity assays in *C. elegans* showed that ZJP treatment (200, 400, and 800 μg/mL) had no significant effects on body length, body width, or reproductive capacity (Figure 1A–C). These results indicate that no detectable toxicity was observed under the tested conditions, supporting its suitability for further functional evaluation. In vitro antioxidant assays showed that ZJP exhibited relatively limited radical scavenging activity against DPPH, ABTS^+^, and hydroxyl radicals, and these data are provided in the Supplementary Information (Figure S1).

### 2.2. ZJP Extends Lifespan in N2 and Transgenic Worms

**Figure 2:**
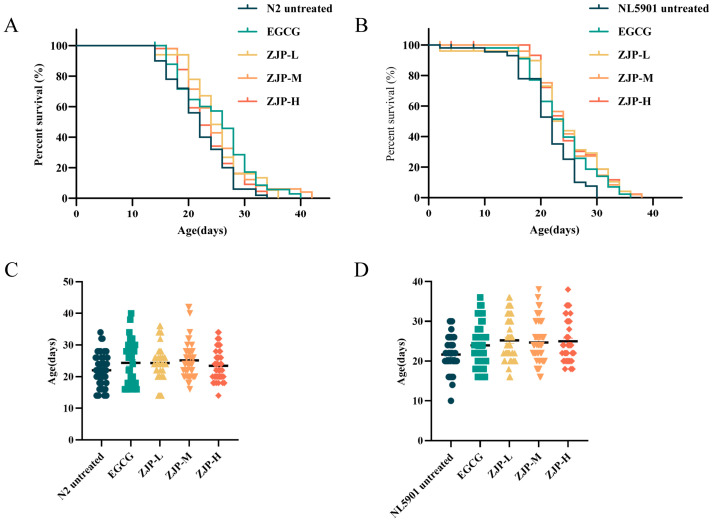
Effects of ZJP on lifespan in *C. elegans*. (**A**) Survival curves of N2. (**B**) Survival curves of NL5901. (**C**) Individual lifespan distribution of N2. (**D**) Individual lifespan distribution of NL5901. Note: *n* = 150 worms per group per experiment. Experiments were performed in three independent biological replicates.

To determine whether ZJP confers a general pro-longevity effect, we performed lifespan assays in wild-type N2 worms. ZJP treatment shifted the survival curves to the right and increased mean lifespan by 10.3–14.1% compared with the controls (Figure 2A), with improved late-life survival. Consistently, ZJP also prolonged lifespan in NL5901 worms, increasing mean lifespan by 9.1% (Figure 2B). Individual lifespan scatter plots showed increases in both median and mean lifespan without an apparent increase in population variability (Figure 2C,D).

### 2.3. ZJP Improves Locomotion and Feeding Behavior

**Figure 3:**
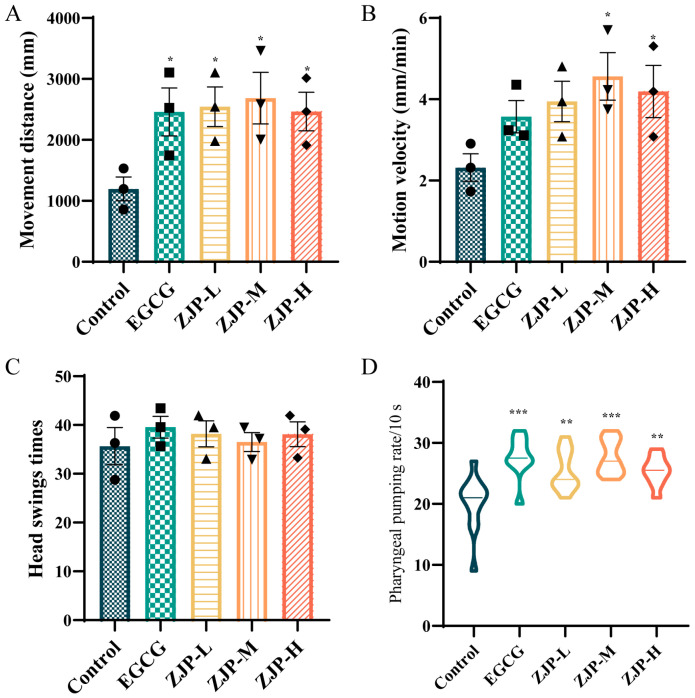
Effects of ZJP on locomotion and feeding behavior in N2 worms. (**A**) Movement distance. (**B**) Locomotion speed. (**C**) Head thrash frequency. (**D**) Pharyngeal pumping rate. Note: *n* = 30 per group, mean ± SD; three independent replicates. Compared with the control, * *p* < 0.05, ** *p* < 0.01, *** *p* < 0.001.

To evaluate the effects of ZJP on neurofunctional decline in N2 worms, locomotor activity and pharyngeal pumping were assessed. ZJP (400 μg/mL) increased locomotion distance and speed by 103.2% and 82.4% (*p* < 0.05 vs. control), respectively, compared with 97.5% and 39.6% in the EGCG group (*p* < 0.05 vs. control) (Figure 3A,B). Head thrash frequency showed no significant difference among groups (Figure 3C), indicating that ZJP may preferentially improve crawling-related locomotor performance rather than all locomotor parameters to the same extent. Pharyngeal pumping increased by 25.6–36.8% following ZJP treatment (*p* < 0.01 vs. control) (Figure 3D). These results indicate that ZJP improves age-associated neuromuscular function in *C. elegans*.

### 2.4. ZJP Reduces Lipofuscin Accumulation

**Figure 4:**
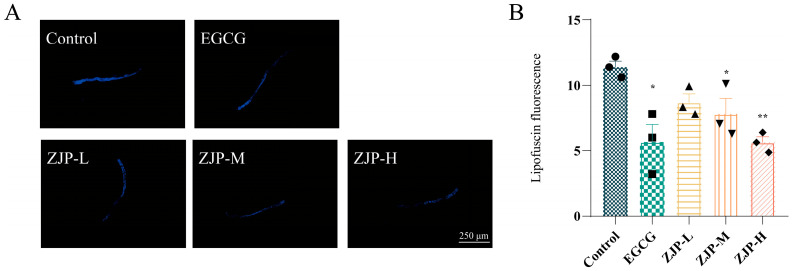
Effects of ZJP on lipofuscin accumulation in *C. elegans*. (**A**) Representative fluorescence images of lipofuscin. (**B**) Quantitative analysis of fluorescence intensity. Note: *n* ≥ 30 worms per group, mean ± SD; three independent replicates. Compared with the control, * *p* < 0.05, ** *p* < 0.01.

To assess whether ZJP attenuates age-related oxidative damage and cellular aging, intestinal lipofuscin autofluorescence was quantified after 8 days of treatment. Compared with the controls, ZJP significantly decreased lipofuscin fluorescence intensity (*p* < 0.05), with reductions of 26.8%, 38.1%, and 50.6% in the ZJP-L, ZJP-M, and ZJP-H groups, respectively (Figure 4). The positive control EGCG reduced lipofuscin accumulation by 47.3% (*p* < 0.05). Notably, the high-dose ZJP group showed the greatest decrease. Since lipofuscin accumulation reflects age-related oxidative damage and cellular aging, these findings suggest that ZJP may alleviate oxidative damage-related lipofuscin accumulation during aging.

### 2.5. ZJP Improves Stress Resistance in C. elegans

**Figure 5:**
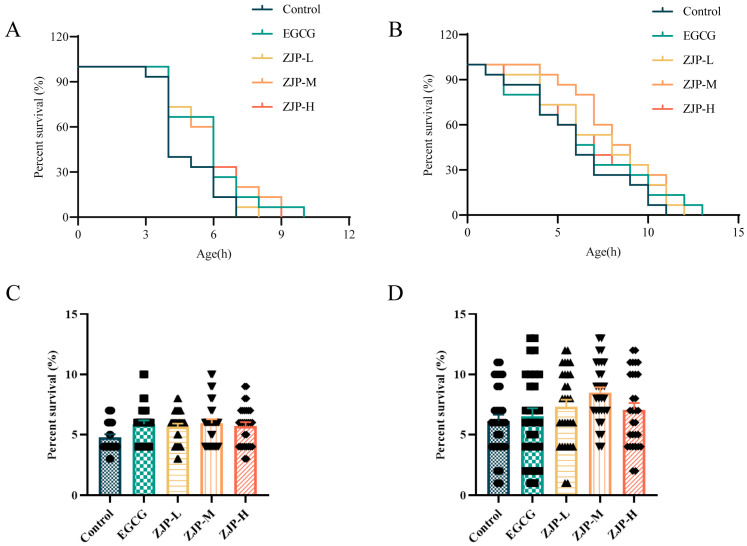
Effects of ZJP on stress survival in N2. (**A**) Survival curves under heat stress (37 °C). (**B**) Survival curves under oxidative stress (H_2_O_2_, 20 °C). (**C**) Mean survival time under heat stress (37 °C). (**D**) Mean survival time under oxidative stress (H_2_O_2_, 20 °C). Note: *n* = 50 worms per group, mean ± SD; three independent replicates.

To determine whether ZJP enhances survival under environmental stress, we subjected N2 worms to heat stress (37 °C) and oxidative stress (H_2_O_2_) after ZJP pretreatment. Each experimental group included 50 worms, with three independent biological replicates. Compared with the control group, ZJP shifted survival curves to the right under both heat stress (Figure 5A) and oxidative stress (Figure 5B), indicating improved stress tolerance. Quantitative analysis showed that the medium dose (ZJP-M) provided the strongest protection, increasing mean survival time by 23.6% under heat stress (Figure 5C) and by 38.1% under oxidative stress (Figure 5D). This non-linear dose–response pattern suggests that the protective effects of ZJP may be dose-dependent, with an optimal effective concentration under the tested conditions. Together, these data indicate that ZJP enhances stress resilience in *C. elegans*, with a particularly pronounced effect against oxidative stress.

### 2.6. ZJP Improves Sensory and Motor Behaviors in Neuroimpairment Models

**Figure 6:**
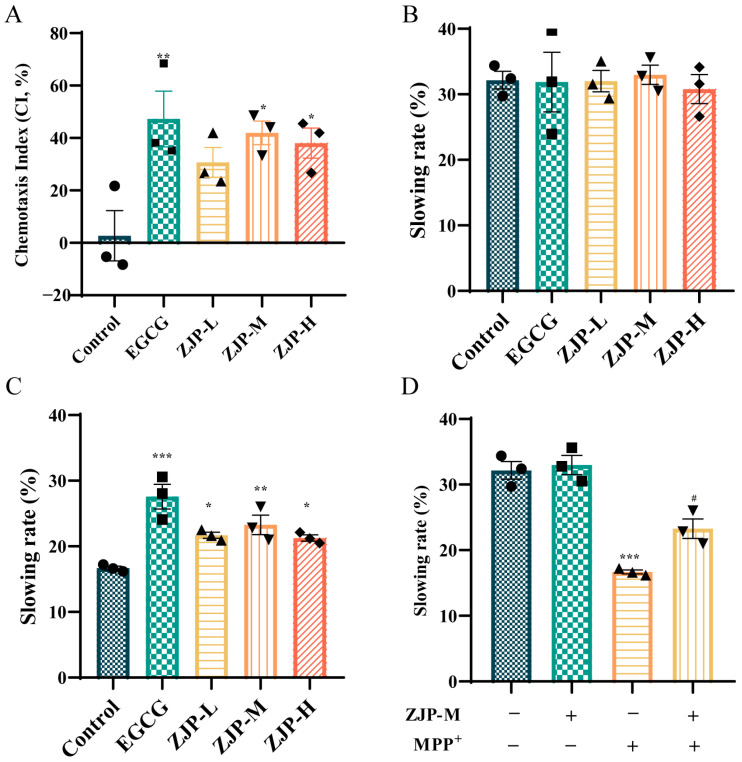
Effects of ZJP on chemotaxis and food-sensing behavior in *C. elegans*. (**A**) Chemotaxis in CL4176. (**B**) Food-sensing behavior in BZ555 without MPP^+^. (**C**) Food-sensing behavior in BZ555 with MPP^+^. (**D**) Percentage change in BZ555 after ZJP treatment relative to the control group. Note: *n* = 50 worms per group, mean ± SD; three independent biological replicates. Compared with the control group, * *p* < 0.05, ** *p* < 0.01, *** *p* < 0.001; Compared with the MPP^+^-treated control, # *p* < 0.05.

To determine whether ZJP alleviates sensory–motor dysfunction, we measured NaCl chemotaxis in CL4176 worms (a neurodegeneration model), and food-induced slowing in BZ555 worms. CL4176 controls showed minimal chemotaxis (CI = 2.7%), whereas EGCG increased CI to 47.3% (Figure 6A). ZJP significantly improved chemotaxis, with CI values of 30.7%, 42.0%, and 38.0% in the ZJP-L, ZJP-M, and ZJP-H groups, respectively (*p* < 0.05 vs. control; Figure 6A). In BZ555 worms, ZJP did not affect food sensing without MPP^+^ (1-methyl-4-phenylpyridinium) (*p* > 0.05; Figure 6B). MPP^+^ reduced food-sensing performance by 48.15% (*p* < 0.05), which was partially restored by ZJP (+39.52% vs. MPP^+^ control, *p* < 0.05; Figure 6C,D). Together, these data indicate that ZJP rescues sensory–motor behavioral deficits in distinct neuro-impairment models.

### 2.7. ZJP Improves Parkinson-Associated Proteotoxicity and Dopaminergic Dysfunction

**Figure 7:**
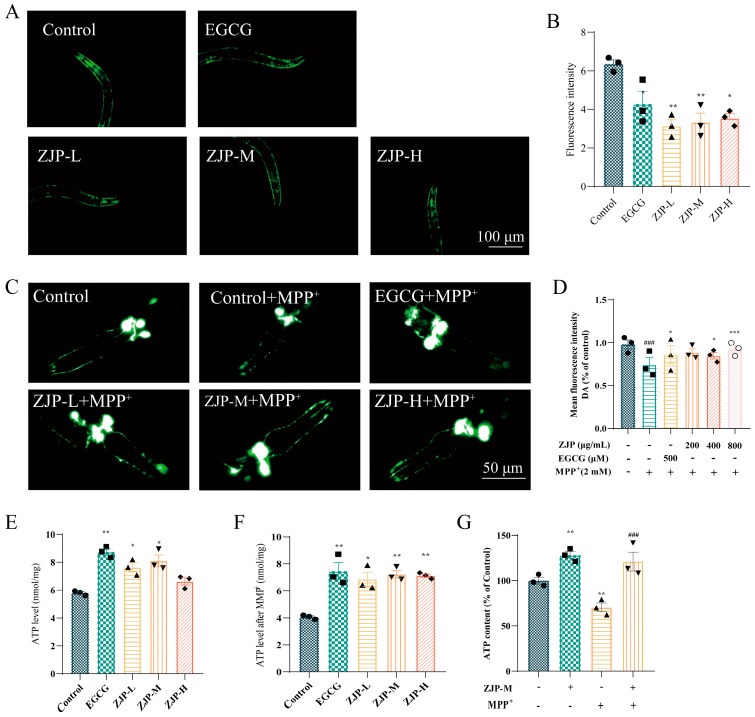
ZJP reduces aggregation-associated α-syn::YFP fluorescence and protects dopaminergic neurons in transgenic worms. (**A**) Representative α-syn fluorescence images (NL5901). (**B**) α-syn fluorescence quantification. (**C**) Representative dopamine (DA) fluorescence images (BZ555). (**D**) DA fluorescence after MPP^+^ (BZ555). (**E**) Adenosine triphosphate (ATP) without MPP^+^. (**F**) ATP with MPP^+^. (**G**) ATP as % of control. Note: *n* ≥ 50 worms per group, mean ± SD; three independent replicates. Compared with the MPP^+^-treated control, ### *p* < 0.001; Compared with the control, * *p* < 0.05, ** *p* < 0.01, *** *p* < 0.001.

To evaluate PD-related outcomes, we assessed α-syn::YFP fluorescence intensity and locomotion in NL5901 worms, as well as dopamine (DA) neuronal integrity and adenosine triphosphate (ATP) levels in MPP^+^-challenged BZ555 worms. In NL5901, 3-day ZJP treatment visibly reduced α-syn::YFP fluorescence intensity compared with the controls (Figure 7A). Quantitative analysis showed fluorescence intensity decreased by 54.9%, 51.1%, and 44.0% in the ZJP-L, ZJP-M, and ZJP-H groups, respectively (*p* < 0.05) (Figure 7B). In BZ555, MPP^+^ exposure markedly reduced GFP fluorescence intensity in CEP dopaminergic neurons (*p* < 0.001), indicating neuronal damage (Figure 7C,D). ZJP significantly restored GFP fluorescence intensity relative to the MPP^+^-treated control (*p* < 0.05) (Figure 7C,D). MPP^+^ exposure reduced ATP levels by 29.64% (*p* < 0.01). Under basal conditions, ZJP-M increased ATP levels by 33.48% (*p* < 0.01). After MPP^+^ challenge, ZJP also restored ATP levels, with ZJP-M group showing a 71.14% increase (*p* < 0.001) (Figure 7E–G). These data suggest that ZJP reduces α-syn-associated proteotoxicity, protects DA neuronal function, and improves energy metabolism in PD *C. elegans* models.

### 2.8. In Vivo Antioxidant Effects of ZJP

**Figure 8:**
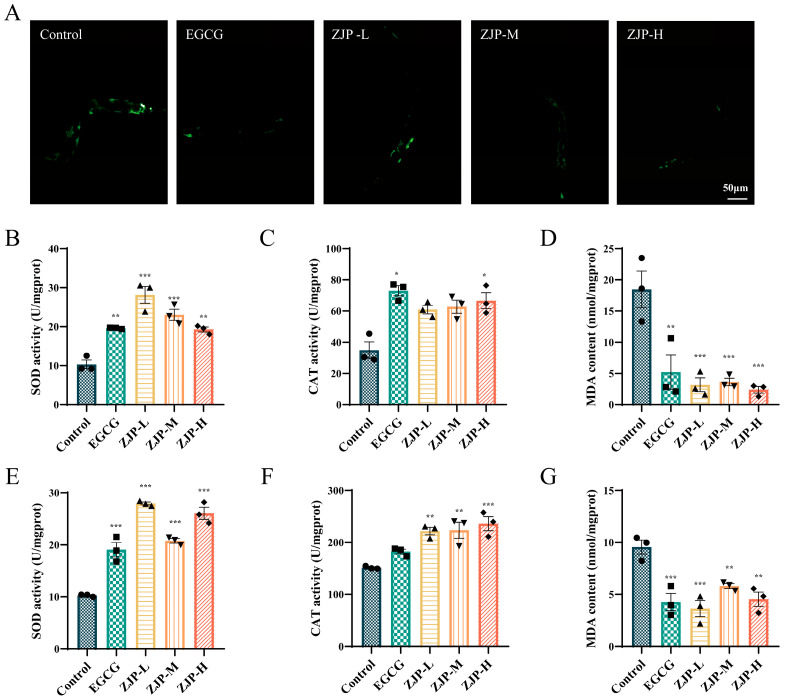
Effects of ZJP on reactive oxygen species (ROS) and antioxidant enzyme activities in *C. elegans*. (**A**) ROS staining in N2. (**B**) superoxide dismutase (SOD) activities in N2. (**C**) catalase (CAT) activities in N2. (**D**) malondialdehyde (MDA) content in N2. (**E**) SOD activities in NL5901. (**F**) CAT activities in NL5901. (**G**) MDA content in NL5901. For ROS staining, *n* = 30 worms per group, mean ± SD; three independent replicates. SOD, CAT, and MDA assays were performed in triplicate. Compared with the control, * *p* < 0.05, ** *p* < 0.01, *** *p* < 0.001.

To assess the in vivo antioxidant effects of ZJP, reactive oxygen species (ROS) levels and antioxidant enzyme activities were measured. H_2_DCFDA staining showed that ROS levels were significantly reduced in ZJP-treated worms, as indicated by decreased fluorescence intensity (Figure 8A). Notably, day 10 worms were selected for ROS measurement to better reflect elevated oxidative stress levels during later stages under the experimental conditions. In N2 worms, ZJP (200 μg/mL) increased superoxide dismutase (SOD) and catalase (CAT) activities (*p* < 0.05), while decreasing malondialdehyde (MDA) levels (Figure 8B–D). In NL5901 worms, SOD and CAT activities increased, with a 61.8% reduction in MDA (*p* < 0.01) (Figure 8E–G). These results indicate that ZJP markedly enhances endogenous antioxidant defenses and mitigates oxidative stress.

### 2.9. ZJP Alters Gene Expression of Signaling Pathways Related to Stress Response

**Figure 9:**
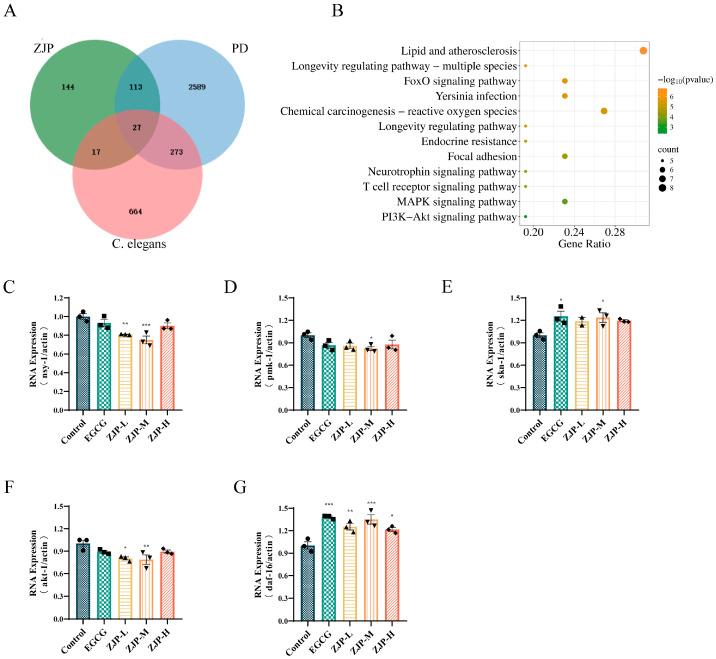
ZJP alters gene expression of signaling pathways related to stress response in *C. elegans*. (**A**) Overlapping targets among ZJP, PD-related genes, and *C. elegans*. (**B**) Kyoto Encyclopedia of Genes and Genomes (KEGG) pathway enrichment of overlapping targets. Relative mRNA expression of genes, including *nsy-1* (**C**), *pmk-1* (**D**), *skn-1* (**E**), *akt-1* (**F**), *daf-16* (**G**). Three independent biological replicates, mean ± SD. Compared with the control, * *p* < 0.05, ** *p* < 0.01, *** *p* < 0.001.

To explore the potential molecular basis of ZJP’s neuroprotective activity, network pharmacology and RT-qPCR analyses were performed. In the network pharmacology analysis, potential targets were predicted based on the monosaccharide components of ZJP, which were used as simplified structural representatives of the complex polysaccharide. A total of 27 overlapping targets were identified among ZJP-related targets, PD-related genes, and *C. elegans* (Figure 9A). Kyoto Encyclopedia of Genes and Genomes (KEGG) enrichment analysis suggested that these targets were mainly associated with antioxidant, longevity pathways (FoxO, MAPK, PI3K-Akt) and neurotransmitter-related pathways (Figure 9B). RT-qPCR analysis further showed that ZJP treatment was associated with changes in the expression of multiple stress-related genes. ZJP significantly downregulated the MAPK genes *nsy-1* and *pmk-1*, while upregulating the antioxidant transcription factor *skn-1* (Figure 9C–E). In addition, ZJP decreased *akt-1* expression and increased *daf-16* expression in the PI3K/Akt/FoxO pathway (Figure 9F,G). These results suggest that the neuroprotective effects of ZJP may be associated with the regulation of stress-response and antioxidant-related signaling pathways.

### 2.10. ZJP Alters the Expression of Genes Related to Neurotransmission

**Figure 10:**
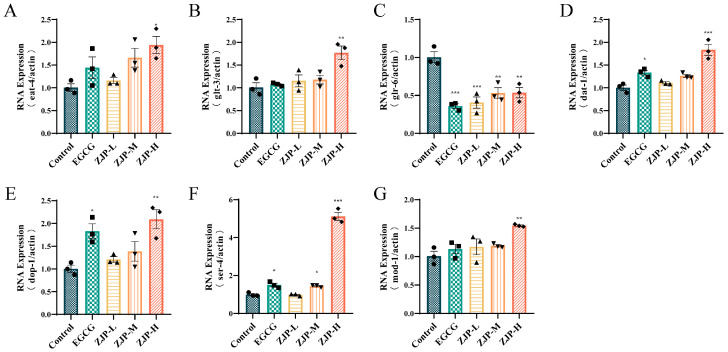
Relative mRNA expression of neurotransmission-related genes, including *eat-4* (**A**), *glt-3* (**B**), *glr-6* (**C**), *dat-1* (**D**), *dop-1* (**E**), *ser-4* (**F**), and *mod-1* (**G**). Three independent biological replicates, mean ± SD. Compared with the control, * *p* < 0.05, ** *p* < 0.01, *** *p* < 0.001.

Moreover, ZJP significantly regulated neurotransmission-related genes, including upregulation of *eat-4* and *glt-3*, downregulation of *glr-6*, and increased expression of *dat-1*, *dop-1*, *ser-4*, and *mod-1* (Figure 10A–G). These findings provide exploratory evidence that ZJP may influence neurotransmission-related molecular pathways and may help explain the observed improvements in PD-related behaviors. In addition, these gene expression changes are consistent with the reduced oxidative stress and enhanced antioxidant capacity observed in ZJP-treated worms.

## 3. Discussion

This study systematically evaluated the physicochemical properties, safety, neuroprotective effects, and potential mechanisms of ZJP in *C. elegans* models of PD and aging. ZJP exhibits both safety and bioactivity, as evidenced by its ability to reduce α-synuclein fluorescence intensity, preserve dopaminergic neuronal integrity, and improve cellular energy status. In addition, it was accompanied by changes in stress-response-related gene expression. These findings collectively suggest that ZJP may represent a potential food-derived candidate for promoting neuroprotection and healthy aging.

In terms of antioxidant activity, ZJP significantly reduced ROS and MDA levels while increasing SOD and CAT activities, indicating an improved intracellular redox state, a pattern consistent with many food-derived polysaccharides. For example, *Lycium barbarum* polysaccharides have been reported to enhance antioxidant enzyme activity and reduce free radical levels through the insulin-growth factor-1 signaling pathway [24,25]. Notably, these antioxidant-related effects were observed in both normal aging worms and PD models, together with reduced α-syn::YFP fluorescence intensity.

On a molecular level, ZJP treatment correlated with transcriptional changes in multiple conserved pathways, including PI3K/Akt/DAF-16, SKN-1, and MAPK. These changes may collectively contribute to the attenuation of oxidative stress and neuronal injury. DAF-16 and SKN-1 are well-characterized transcription factors involved in insulin/IGF-1 signaling, oxidative stress resistance, and lifespan in *C. elegans*, as established in classical aging studies [26,27]. In contrast to previous investigations that largely target a single predominant pathway, our findings suggest that ZJP may engage parallel protective networks. For example, the *Polygonatum sibiricum* polysaccharide has been reported to ameliorate cognitive deficits in Alzheimer’s disease models via gut microbiota remodeling along the gut–brain axis [28], whereas *Lycium barbarum* polysaccharides confer protection against glaucomatous damage mainly through suppression of the RAGE/Aβ pathway [29]. Although such pathway-focused mechanisms also exhibit substantial neuroprotective potential, the apparent multi-target profile of ZJP may be particularly relevant to the multifactorial pathology of neurodegeneration and may help support overall neuronal homeostasis. Additionally, ZJP treatment modulates the expression of neurotransmitter-related genes. The upregulation of *eat-4* and *glt-3* together with the downregulation of *glr-6* may indicate changes in glutamatergic neurotransmission-related gene expression. Meanwhile, the increased expression of *dat-1*, *dop-1*, *ser-4*, and *mod-1* suggests that dopaminergic and serotonergic neurotransmission-related genes may also be affected by ZJP treatment. Notably, these observations are based on mRNA changes, and no direct analyses of neurotransmitter levels, synaptic transmission, or neuronal activity were performed. Therefore, these results should be interpreted as indirect evidence of neurotransmission-related gene regulation rather than direct functional evidence of neurotransmission modulation. Nevertheless, they provide transcriptional clues that may help explain the sensory–motor behavioral changes observed after ZJP treatment.

With respect to behavior and lifespan parameters, ZJP improved locomotion speed and pharyngeal feeding frequency, and extended the lifespan in both normal and PD-model worms, with enhanced tolerability to heat and oxidative stress. These effects are consistent with previous reports showing that plant-derived polysaccharides can attenuate aging-related functional decline and improve locomotor performance [30]. Importantly, ZJP exhibited neuroprotective effects across both genetic and toxin-induced PD models, supporting its candidacy as a food-derived neuroprotective agent. Interestingly, a consistent pattern was observed across multiple assays in which the medium dose of ZJP yielded the most pronounced effects. This non-linear dose–response relationship may indicate an optimal effective concentration under the tested conditions. Possible explanations include a hormetic response at moderate concentrations, saturation of uptake or biological responses at higher concentrations, or differential metabolic responses to different ZJP doses.

Collectively, these results support a working model in which ZJP altered expression of genes involved in PI3K/Akt/DAF-16, SKN-1, and MAPK stress-response signaling pathways. Importantly, because our pathway evidence is primarily based on transcriptional readouts, these associations should be interpreted as mechanistic correlates rather than definitive causal links. The concurrent improvements in ATP levels and reduction in oxidative stress are consistent with a cellular environment that is less permissive to α-synuclein proteotoxicity, which may in turn contribute to the preservation of dopaminergic neuron function and behavioral performance.

From a functional food-development perspective, ZJP-derived from jujube, a commonly consumed food with an established safety profile, may represent a potential candidate for further development as a neuroprotective dietary component [31]. However, these preliminary findings require further validation in mammalian models for direct translational applications.

This study has several limitations. The findings from *C. elegans* require validation in mammalian PD models. The proposed mechanisms were mainly derived from transcriptional data without functional validation using mutant strains, pathway inhibition, or protein-level analyses. In addition, the network pharmacology analysis was preliminary, and monosaccharide-based prediction do not fully reflect the structural complexity of intact ZJP. Lastly, the structure-activity relationship and bioavailability of ZJP remain to be clarified.

## 4. Materials and Methods

### 4.1. Materials

The following materials were used: H2DCFDA (UEland, Hangzhou, China); SOD, CAT, and MDA ELISA kits (Nanjing Jiancheng, Nanjing, China); MPP^+^ (1-methyl-4-phenylpyridinium; ANPEL, Shanghai, China; 730264); Epigallocatechin gallate (EGCG; Macklin, Shanghai, China; E8120); CellTiter-Glo^®^ Luminescent Cell Viability Assay (Promega, Madison, WI, USA, G7570); BCA protein assay kit (Beyotime, Shanghai, China; P0010S); RNA extraction kit (Omega Bio-tek, Norcross, GA, USA; R6834-01); SYBR Green qPCR Master Mix (Yeasen, Shanghai, China; 11201ES50); and One-Step RT SuperMix with gDNA Remover for qPCR (Yeasen, Shanghai, China; 11142ES60).

The *C. elegans* strains used in this study included N2, CL4176, NL5901, and BZ555. The wild-type N2 strain was used as the basic control strain and for evaluating the safety, lifespan-extending effect, stress resistance, locomotor function, pharyngeal pumping, chemotaxis, lipofuscin accumulation, and oxidative stress-related indicators of ZJP. CL4176 (dvIs27X), which expresses human Aβ in muscle cells, was used as an Aβ-associated proteotoxicity model to evaluate paralysis induced by temperature-dependent Aβ expression. NL5901 [*unc-54p*::α-synuclein::YFP + unc-119(+)], which expresses human α-synuclein fused with yellow fluorescent protein (α-syn::YFP) in body wall muscle cells, was used as a transgenic PD-related proteotoxicity model to assess α-syn::YFP fluorescence accumulation. BZ555 [*egIs1*(*dat-1p*::GFP)], which expresses GFP in dopaminergic neurons under the control of the *dat-1* promoter, was used to evaluate dopaminergic neuronal integrity. In addition, BZ555 worms treated with MPP^+^ were used as a toxin-induced PD-like model. These strains are widely used for studying neurodegeneration, protein aggregation, and dopaminergic neuron function. All worms were maintained on nematode growth medium (NGM) plates seeded with *Escherichia coli* OP50 (used as the food source) at 20 °C under standard laboratory conditions.

### 4.2. Preparation and Compositional Analysis of ZJP

The extraction, purification, and structural characterization of ZJP have been reported in our previous study [32]. Briefly, ZJP was obtained from *Ziziphus jujuba* cv. Huizao using hot water extraction, followed by ethanol precipitation, deproteinization, decolorization, dialysis, and DEAE-52 column purification. As previously reported [30], the purified polysaccharide contained 92.7% total sugars and 0.47% protein, with an extraction yield of 5.2%. The monosaccharide composition is summarized in Table 1.

The structural characteristics of ZJP have been described in our previous study [32]. ZJP was identified as a weakly acidic, amorphous, and thermally stable branched heteropolysaccharide. It was mainly composed of glucose, and contained →6)-β-D-glucopyranosyl,→3)-β-D-galactopyranosyl, and terminal α-L-rhamnopyranosyl/α-L-arabinofuranosyl residues. The purity of ZJP was further confirmed by HPGPC, showing a single symmetrical peak, with an average molecular weight of approximately 66.6 kDa [32]. Representative chromatograms and spectra are provided in the Supplementary Information (Figures S2–S6).

### 4.3. Antioxidant Activity of ZJP In Vitro

The antioxidant activity of ZJP was evaluated using three radical scavenging assays following established protocols. For the DPPH assay, ZJP solution was mixed with 0.1 mg/mL DPPH ethanolic solution (1:1, *v*/*v*), incubated in the dark for 30 min at room temperature, and absorbance was measured at 517 nm [33]. The ABTS^+^ assay was performed by mixing 0.1 mL of ZJP solution with 4.9 mL of ABTS working solution, incubated at room temperature for 10 min, and absorbance was recorded at 734 nm [34]. The hydroxyl radical scavenging activity was assessed by combining 1 mL of ZJP solution with 1 mL of hydroxyl radical-generating solution, incubating at 37 °C for 30 min, and measuring absorbance at 510 nm [35]. All in vitro antioxidant assays were performed in triplicate.

### 4.4. Toxicity Assessment

#### 4.4.1. Synchronization of *C. elegans*

Synchronized *C. elegans* populations were obtained by treating gravid adults with an alkaline hypochlorite solution, which dissolves the adults and larvae while leaving the eggs intact. The collected eggs were incubated in M9 buffer and allowed to hatch and develop to the L4 stage [19].

#### 4.4.2. Acute Toxicity

Worms were exposed to ZJP (10–2000 μg/mL), with EGCG (500 μM, a natural antioxidant polyphenol) as the positive control [36]. Acute toxicity was assessed by exposing 30 synchronized L4-stage *C. elegans* per group to 200 μL of solutions in 96-well plates for 24 h [37]. Survival was recorded microscopically, and worms were considered dead if unresponsive to stimulation within 10 s. Experiments were conducted in triplicate. No obvious toxicity was observed across the tested range (10–2000 μg/mL); therefore, concentrations of 200, 400, and 800 μg/mL were selected for subsequent functional assays.

#### 4.4.3. Developmental Toxicity

Synchronized L4-stage *C. elegans* were transferred to NGM plates and divided into five groups: control (untreated), positive control (EGCG, 500 μM), and ZJP-treated groups at low (ZJP-L, 200 μg/mL), medium (ZJP-M, 400 μg/mL), and high (ZJP-H, 800 μg/mL) doses. After 3 days, worms were anesthetized with 10 mM levamisole, mounted on 2% agar pads on glass slides, and covered with a coverslip for microscopy. Body length and width were then measured under a light microscope [19]. Thirty worms per group were analyzed in each experiment, and three independent experiments were performed.

#### 4.4.4. Reproductive Toxicity

Synchronized L4-stage *C. elegans* were placed individually on NGM plates containing control or ZJP, with one worm per plate and ten worms in each group. *C. elegans* were moved to fresh plates until reproduction ceased, and progeny were counted 48 h later [19]. This assay was performed in three independent experiments.

### 4.5. Neuroprotective Function Assays

#### 4.5.1. Lifespan Assay

Synchronized *C. elegans* were randomly assigned to NGM plates and divided into five groups: control, positive control (EGCG, 500 μM), and ZJP groups (200, 400, and 800 μg/mL). To prevent progeny interference, 12.5 mg/mL 5-FU was added to minimize confounding by offspring production during survival analysis. Survival was recorded daily [38]. For each group, 150 worms were used per experiment (50 worms per plate, three plates), and three independent biological replicates were performed. Survival data were analyzed using the Kaplan–Meier method and compared by the log-rank test.

#### 4.5.2. Locomotion Assay

On day 3 of adulthood, synchronized worms from each treatment group (control, positive control with EGCG 500 μM, and ZJP at 200, 400, and 800 μg/mL) were placed on fresh NGM plates. Locomotor behavior was recorded for 30 s using a self-developed worm locomotion recording and analysis system. Movement distance, locomotion speed, and head thrash frequency were all automatically quantified from the recorded trajectories by the software [39]. Pharyngeal pumping was assessed by counting pharyngeal contractions within 10 s under a microscope. Thirty worms per group were analyzed in each experiment, with three independent replicates.

#### 4.5.3. Oxidative and Thermal Stress Tolerance

*C. elegans* were pretreated with ZJP (200, 400, and 800 μg/mL) and EGCG for 3 days. For oxidative stress, pretreated worms were exposed to 4 mmol/L H_2_O_2_ in M9 buffer, and mortality was recorded hourly. For thermal stress, worms were incubated at 37 °C and deaths were recorded every 2 h. Worms unresponsive to touch with a straight body were considered dead [40]. Fifty worms per group were used for each assay, and all experiments were repeated three times independently.

#### 4.5.4. ROS Measurement

ROS levels were determined as described previously [40]. Day 10, N2 worms, treated with or without ZJP, were stained with 25 μM H_2_DCF-DA in the dark for 30 min, washed three times with M9 buffer to remove excess dye, and anesthetized with 10 mM levamisole. Worms were mounted on 2% agar pads on glass slides, covered with a coverslip, and immediately imaged under a fluorescence microscope (Ex 485 nm, Em 525 nm) using identical exposure settings for all groups. Fluorescence intensity was quantified from the whole worm using ImageJ software (version 1.53t). Background fluorescence was subtracted, and data were normalized to the control group. Thirty worms per group were analyzed in three independent experiments.

#### 4.5.5. Antioxidant Enzyme Activity

N2 worms treated with ZJP (200, 400, and 800 μg/mL, 3 d) were analyzed for SOD, CAT, and MDA levels using commercial ELISA kits. Total protein content was quantified using the BCA method. Results were normalized to this value and expressed as units per milligram of protein. All assays were performed in triplicate.

#### 4.5.6. Lipofuscin Accumulation

Lipofuscin accumulation was assessed in day 8 N2 worms treated with ZJP (200, 400, and 800 μg/mL). Worms were anesthetized with 10 mM levamisole, mounted on 2% agar pads on glass slides, covered with a coverslip, and intestinal autofluorescence was imaged under blue excitation and quantified with ImageJ software (version 1.53t) [41]. At least 30 worms per group were analyzed from three independent experiments.

### 4.6. Neurodegenerative Disease Models

#### 4.6.1. Chemotaxis Assay

NaCl gradients were prepared on 9 cm NaCl-free NGM plates by placing an agar block containing 100 mM NaCl. The block was removed before testing. After 3-day treatments, synchronized L4 worms were starved for 4 h on NaCl-containing, OP50 free NGM plates. Worms were placed at the plate center, levamisole was added to regions A and B, and worms in each region were counted after 30 min at 20 °C. The chemotaxis index (CI) was calculated as (number of worms in region A − number of worms in region B) divided by the total number of worms. Fifty worms per group were analyzed in each experiment, with three biological replicates.

#### 4.6.2. Fluorescence Imaging of α-Synuclein::YFP and Dopaminergic Neurons

For α-synuclein aggregation analysis, synchronized L4-stage NL5901 worms were treated with ZJP for 3 d, anesthetized with 10 mM levamisole, mounted on 2% agar pads on glass slides, and imaged under a fluorescence microscope at 488 nm [42]. Separately, BZ555 were exposed to 2 mM MPP^+^ (PD-related neurotoxin) for 1 h, washed three times with M9 buffer, and subsequently treated with ZJP for 2 d. Worms were then anesthetized, mounted on agar pads on glass slides, and GFP fluorescence in CEP dopaminergic neurons was imaged and quantified using ImageJ software (version 1.53t) [43]. Images were acquired under identical settings, background fluorescence was subtracted, and data were normalized to the corresponding control group. GFP fluorescence intensity in CEP dopaminergic neurons was quantified using the same ImageJ software as a measure of neuronal integrity. At least 50 worms per group were imaged and analyzed in three independent experiments.

#### 4.6.3. Food-Sensing Behavior

Assessment of food-sensing behavior in BZ555 worms was performed following a previously described protocol [44]. BZ555 worms, with or without MPP^+^ pretreatment (2 mM, 1 h), were cultured on control or ZJP plates (200, 400, and 800 μg/mL) for 2 d. Locomotion was measured on NGM with or without OP50, and the slowing rate was calculated. Fifty worms per group were tested in each experiment, with three independent biological replicates.

#### 4.6.4. Adenosine Triphosphate (ATP) Content

BZ555 worms were treated with ZJP or the control for 3 days at 25 °C. After washing with M9 buffer, 50 worms per group were placed into a 96-well plate. CellTiter-Glo reagent (50 μL) was added, and the plate was shaken for 10 min followed by 10 min incubation. Luminescence was measured using a microplate reader (Shanghai Flash Spectrum Biotechnology, Shanghai, China). Total protein content was measured by BCA assay, and ATP levels were normalized to protein. Measurements were performed in triplicate.

### 4.7. Network Pharmacology Analysis

Because ZJP is a complex polysaccharide, its monosaccharide components were used as simplified representatives for target prediction. This analysis served as a preliminary screening approach to provide candidate pathway-level clues for subsequent gene expression validation. Potential targets of ZJP monosaccharides were predicted using TCMSP (https://www.tcmsp-e.com, accessed on 8 October 2025), PubChem (https://pubchem.ncbi.nlm.nih.gov/, accessed on 8 October 2025), PharmMapper (https://www.lilab-ecust.cn/pharmmapper/index.html, accessed on 8 October 2025), and standardized in UniProt (https://www.uniprot.org/, accessed on 9 October 2025). Target proteins of *C. elegans* were retrieved from UniProt. PD-related targets were retrieved from DisGeNET (https://disgenet.com/, accessed on 10 October 2025), OMIM (https://www.omim.org/, accessed on 10 October 2025), and GeneCards (https://www.genecards.org/, accessed on 10 October 2025), and overlapping targets were identified with jvenn (https://jvenn.toulouse.inra.fr/app/example.html, accessed on 11 October 2025). KEGG enrichment was performed in DAVID (https://davidbioinformatics.nih.gov/, accessed on 11 October 2025) to explore pathways involved in ZJP intervention in PD [45].

### 4.8. Gene Expression Analysis

Synchronized L4-stage *C. elegans* were divided into control, EGCG, 500 μM, and ZJP groups (200, 400, and 800 μg/mL). Worms were cultured on NGM for 3 d, then collected into sterile RNase-free tubes. Total RNA was extracted using an RNA extraction kit, reverse-transcribed into cDNA with a Yeasen kit, and target gene expression was quantified by RT-qPCR. Primer sequences are listed in Table S1. Three independent biological replicates were analyzed.

### 4.9. Statistical Analysis

Statistical analysis was carried out with GraphPad Prism 8.3. Unless otherwise stated, each experiment was performed in three independent biological replicates, and data from these replicates were combined for statistical analysis rather than presented as representative experiments. Results are presented as mean ± SD. Before one-way ANOVA, data normality and homogeneity of variance were assessed. Multiple group comparisons were made using one-way ANOVA followed by Tukey’s post hoc test when these assumptions were met, while the Kaplan–Meier method and log-rank test were used for survival data. Fluorescence intensity was quantified using ImageJ software (version 1.53t), and statistical significance was defined as *p* < 0.05.

## 5. Conclusions

This study demonstrates that ZJP is a safe, food-derived polysaccharide with neuroprotective effects in *C. elegans* models of PD and aging. ZJP reduced α-syn::YFP fluorescence, protected dopaminergic neuronal integrity, enhanced antioxidant defenses, improved locomotion and feeding behavior, and extended healthspan. These effects were accompanied by transcriptional changes consistent with alterations in PI3K/Akt/DAF-16, SKN-1, and MAPK signaling pathways, as well as neurotransmission-related genes. Collectively, these findings suggest that ZJP may represent a potential candidate for further investigation in neuroprotection. However, these observations are based on *C. elegans models*, and further validation in mammalian systems, together with detailed mechanistic studies, is required.

## Figures and Tables

**Table 1:** Monosaccharide composition of ZJP.

S/N	Mol ID	Molecule Name	MF	MW	OB (%)	Pubchem ID
1	MOL007677	L-arabinose	C_5_H_10_O_5_	150.13	31.89	5460291
2	MOL002682	D-galactose	C_6_H_12_O_6_	180.16	47.81	3037556
3	MOL000734	D-glucose	C_6_H_12_O_6_	180.16	24.44	107526
4	MOL000420	D-xylose	C_5_H_10_O_5_	150.13	51.08	644160
5	MOL000051	D-mannose	C_6_H_12_O_6_	180.16	1.76	161658
6	MOL004690	Rhamnose	C_6_H_12_O_5_	164.16	40.73	19233
7	MOL000386	L-fucose	C_6_H_12_O_5_	164.16	42.51	3034656
8	MOL000384	DL-glucuronic acid	C_6_H_10_O_7_	194.14	3.35	65041
9	MOL000383	D-galacturonic acid	C_6_H_10_O_7_	194.14	29.75	84740

Note: S/N, serial number; Mol ID, molecule identifier; MF, molecular formula; MW, molecular weight; OB (%), oral bioavailability percentage; Pubchem ID, pubchem compound identifier.

## Data Availability

The original contributions presented in this study are included in the article/Supplementary Materials. Further inquiries can be directed to the corresponding author.

## Supplementary Materials

The following supporting information can be downloaded at https://www.mdpi.com/article/10.3390/ijms27114727/s1.
