# Supplementary material for: Jujube Polysaccharide Promotes Neuroprotection and Longevity in Caenorhabditis elegans Through Oxidative Stress Resistance and Stress-Response Signaling
Source: Int J Mol Sci. 2026 May 24;27(11):4727. doi: 10.3390/ijms27114727 (PMC13257073; doi:10.3390/ijms27114727)
Supplement: Supplementary file 1 [file ijms-27-04727-s001.zip › ijms-4311660-supplementary.pdf]

SUPPLEMENTARY INFORMATION

Jujube polysaccharide promotes neuroprotection and longevity in *Caenorhabditis elegans* through oxidative stress resistance and stress-response signaling

Zhiying Hou [1,2], Ayaz Ahmed [1], Jiayin Wang [1], Meng Sun [1], Fengzhong Wang [1], and Qiong Wang [1,2,*]

[1] Institute of Food Science and Technology, Chinese Academy of Agricultural Sciences, Beijing 100193, China; 82101231155@caas.cn (Z.H.); ayaz.ahmed@iccs.edu (A.A.); wangjiayinss@163.com(J.W.);mengsun13140130@163.com(M.S.); wangfengzhong@caas.cn (F.W.); wangqiong01@caas.cn (Q.W.)

[2] National Nanfan Research Institute (Sanya), Chinese Academy of Agricultural Sciences, Sanya 572024, China

[*] Correspondence: wangqiong01@caas.cn (Q.W.)

Table S1 The sequences of qPCR primers

Gene name	Primer sequence
Caenorhabditis elegans actin-1	F: GCTGGACGTGATCTTACTGATTACC
	R: GTAGCAGAGCTTCTCCTTGATGTC
Caenorhabditis elegans nsy-1	F: ACTCTTGGCTCCGACCAATTT
	R: GTGGCAAAGGACACACATCA
Caenorhabditis elegans pmk-1	F: TCACGATGAAACTGATGAGCCA
	R: GCAAATGCCACGTTTTTCTGG
Caenorhabditis elegans skn-1	F: ATTCGTCGACGCGGAAAGAA
	R: GGCTTTAATAAGGTTTCGACCGAG
Caenorhabditis elegans daf-16	F: CGTTTCCTTCGGATTTCA
	R: ATTCCTTCCTGGCTTTGC
Caenorhabditis elegans akt-1	F: GGACAACCGTTTCCTGAG
	R: GACGAACTTCTGCCGACT
Caenorhabditis elegans eat-4	F: TCTTATTAGCCAGTCTTATTCAC
	R: GACCATTCTTCCTCCTCTT
Caenorhabditis elegans glt-3	F: CGTTGCCAGTAACATTCC
	R: TTCCGTCCATTGTAATTGTG
Caenorhabditis elegans glr-6	F: ACCAATCACGAAGGAGTT
	R: TGCCAACACGAGTAAGAT
Caenorhabditis elegans ser-4	F: CTTGTTGGTCGGAATGATAG
	R: CCAATATCGATCCAGAGCAACAA
Caenorhabditis elegans mod-1	F: ATTATTCAAGCCTATGTTCCAA
	R: GAGATGAGATTCCGACAGT
Caenorhabditis elegans dat-1	F: ATGGTAATTGGAATTGCTATGT
	R: CATTGCTTCACAGAACACT
Caenorhabditis elegans dop-1	F: GACGAGCATTCAAGAAGATT
	R: ATTGTCAGGAGCAGCATA

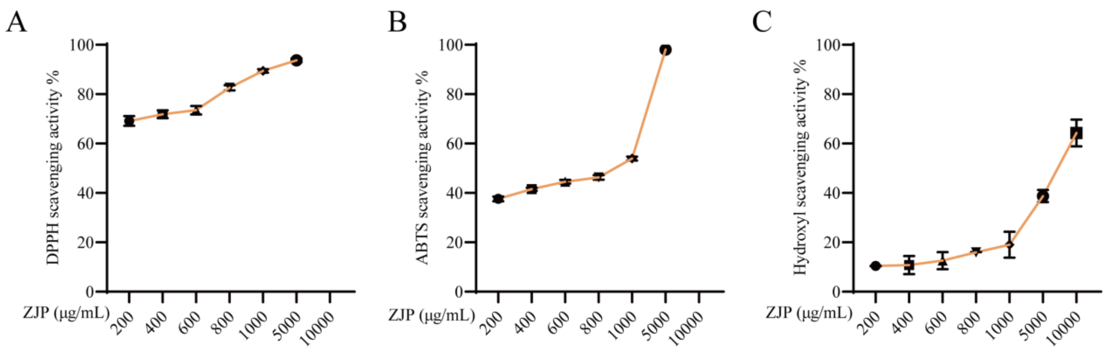

Figure S1. *In vitro* radical scavenging activity of ZJP. (A) DPPH radical scavenging activity of ZJP. (B) ABTS radical scavenging activity of ZJP. (C) Hydroxyl radical scavenging activity of ZJP.

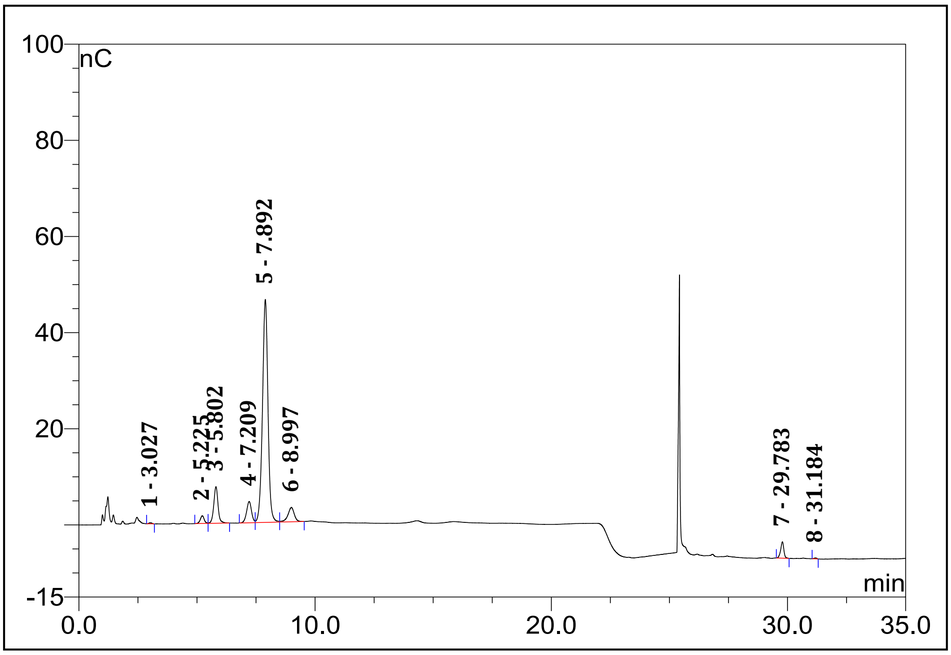

Figure S2. Monosaccharide composition of ZJP. 1, fucose; 2, rhamnose; 3, arabinose; 4, galactose; 5, glucose; 6, xylose; 7, galacturonic acid; 8, glucuronic acid.

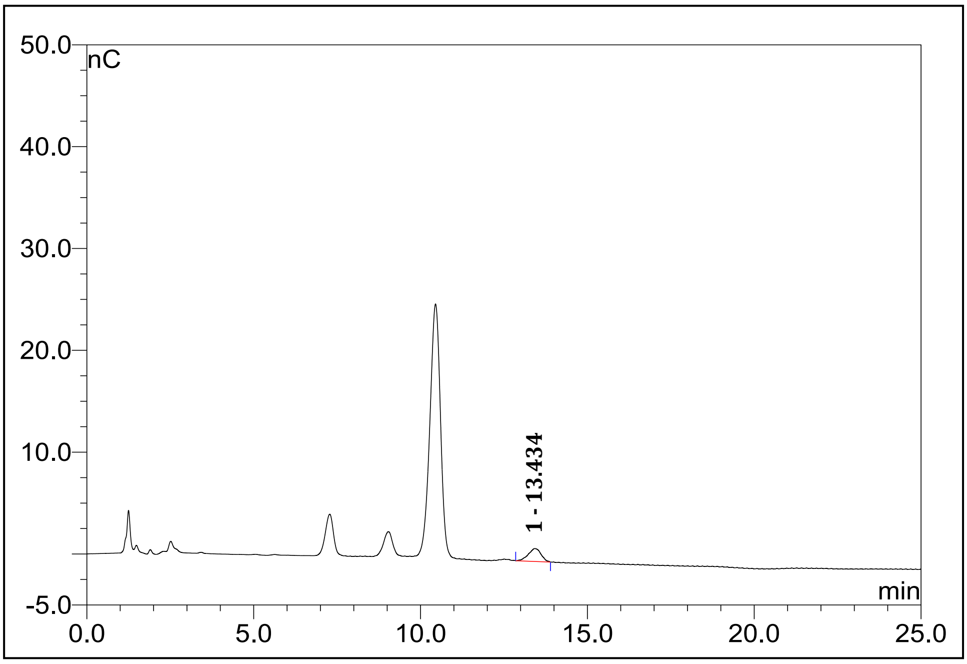

Figure S3. Monosaccharide composition of ZJP. 1, mannose.

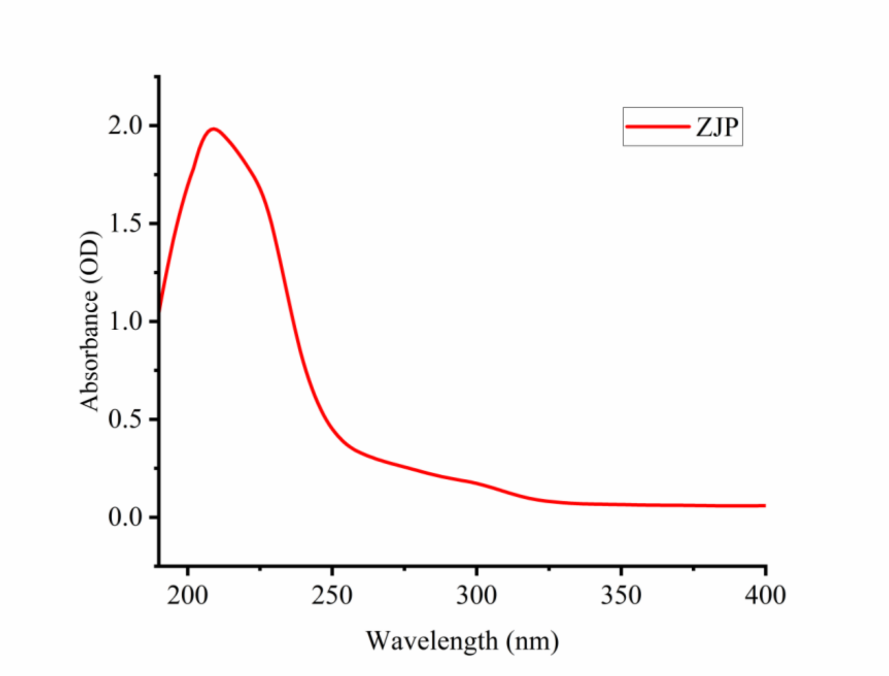

Figure S4. UV spectrum of ZJP.

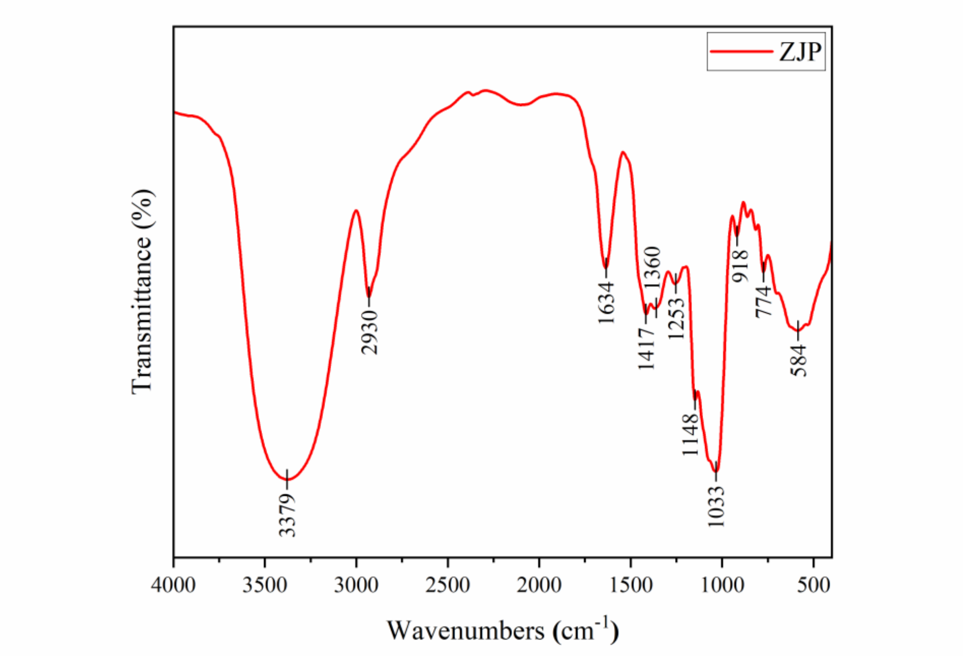

Figure S5. FTIR spectrum of ZJP.

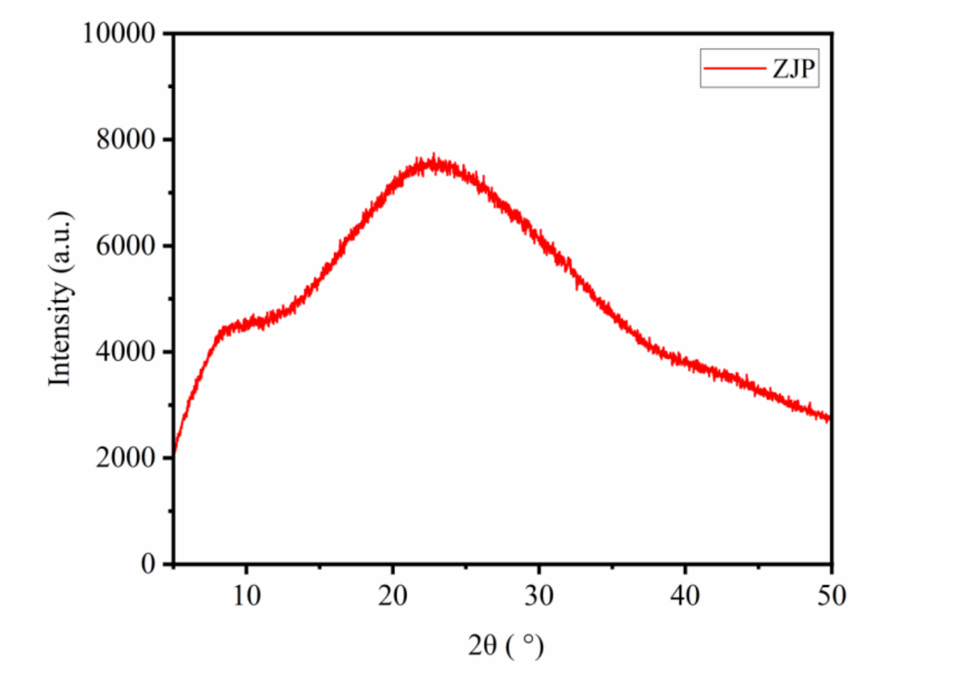

Figure S6. XRD pattern of ZJP.
